# Lung behavior during a staircase high-frequency oscillatory ventilation recruitment maneuver

**DOI:** 10.1186/s40635-024-00623-w

**Published:** 2024-04-25

**Authors:** Pauline de Jager, Alette A. Koopman, Dick G. Markhorst, Martin C. J. Kneyber

**Affiliations:** 1 Division of Paediatric Critical Care Medicine, Department of Paediatrics, Beatrix Children’s Hospital, University Medical Center Groningen, Internal Post Code CA 80, P.O. Box 30.001, 9700 RB Groningen, The Netherlands; 2 Department of Paediatric Intensive Care, UMC, Amsterdam, The Netherlands; 3 Critical Care, Anaesthesiology, Peri-Operative Medicine and Emergency Medicine, The University of Groningen, Groningen, The Netherlands

**Keywords:** High-frequency oscillatory ventilation, Pediatric acute respiratory distress syndrome, Recruitment, Time constant

## Abstract

**Background:**

Lung volume optimization maneuvers (LVOM) are necessary to make physiologic use of high-frequency oscillatory ventilation (HFOV), but lung behavior during such maneuvers has not been studied to determine lung volume changes after initiation of HFOV, to quantify recruitment versus derecruitment during the LVOM and to calculate the time to stabilization after a pressure change.

**Methods:**

We performed a secondary analysis of prospectively collected data in subjects < 18 years on HFOV. Uncalibrated respiratory inductance plethysmography (RIP) tracings were used to quantify lung recruitment and derecruitment during the LVOM inflation and deflation. The time constant was calculated according to the Niemann model.

**Results:**

RIP data of 51 subjects (median age 3.5 [1.7–13.3] months) with moderate-to-severe pediatric acute respiratory distress syndrome (PARDS) in 85.4% were analyzed. Lung recruitment and derecruitment occurred during the LVOM inflation phase upon start of HFOV and between and within pressure changes. At 90% of maximum inflation pressure, lung derecruitment already started during the deflation phase. Time to stable lung volume (time constant) could only be calculated in 26.2% of all pressure changes during the inflation and in 21.4% during the deflation phase, independent of continuous distending pressure (CDP). Inability to calculate the time constant was due to lack of stabilization of the RIP signal or no change in any direction.

**Conclusions:**

Significant heterogeneity in lung behavior during a staircase incremental–decremental LVOM occurred, underscoring the need for higher initial inflation pressures when transitioning from conventional mechanical ventilation (CMV) and a longer time between pressure changes to allow for equilibration.

**Supplementary Information:**

The online version contains supplementary material available at 10.1186/s40635-024-00623-w.

## Background

High-frequency oscillatory ventilation (HFOV) is commonly used in pediatric acute respiratory distress syndrome (PARDS) when oxygenation targets cannot be achieved with conventional mechanical ventilation (CMV) setting below injurious thresholds [1]. The combination of delivering small stoke volumes (1–2 mL/kg) and minimal pressure swings at alveolar level renders HFOV an attractive approach to minimize repetitive alveolar opening and closure and (regional) tidal hyperinflation.

To make use of the potentially advantageous effects of HFOV, it is important to apply an open-lung approach by performing a lung volume optimization maneuver (LVOM) as has been recommended by international pediatric guidelines [2, 3]. The rationale behind this is the direct relationship between lung aeration and oxygenation and that pressures swings are better attenuated in a recruited lung [4, 5]. Aside from this, performing an LVOM allows for oscillating on the deflation limb of the pressure–volume relationship, making use of lung hysteresis [6, 7]. Inappropriate lung aeration increases adverse events and cardiovascular compromise due to either overdistension or atelectasis. Previously, we have shown that an open-lung approach for pediatric HFOV is feasible and safe in terms of oxygenation, ventilation and hemodynamics [8].

Although to our best of knowledge no LVOM has to be shown to be superior to the other, we have implemented in our clinical algorithm a staircase incremental–decremental continuous distending pressure (CDP) titration that allows us to map the quasi-static pressure–volume relationship, with the purpose to identify the optimal CDP on the deflation limb [6–8]. This mapping is guided by transcutaneous oxygen saturation (SpO_2_) and/or fraction of inspired oxygen (FiO_2_) as markers of lung recruitment, although previously we reported that these metrics may not adequately reflect lung aeration, underscoring the requirement of monitoring techniques [9, 10]. Furthermore, there a several challenges when transitioning a subject from CMV of HFOV, including what the initial CDP should be and how rapidly lung volume stabilizes after pressure changes during the LVOM. The time required to achieve a new equilibrium after pressure changes is determined by the time constant, but so far no pediatric studies that have examined this except for one study in pediatric subjects on conventional mechanical ventilation where it was observed that changes in dynamic pulmonary compliance occurred after more than 30 min and oxygenation after more than 1 h following an incremental PEEP change and related to lung injury severity [11].

A better understanding of lung behavior following pressure changes during an LVOM has important clinical implications as adequate time must be allowed for stabilization of the newly aerated lung areas. In a prospectively collected dataset of subjects with moderate-to-severe lung injury who were transitioned from CMV to HFOV, we therefore sought to determine (a) the response in lung volume changes immediately after initiation of HFOV, (b) to quantify recruitment versus derecruitment during the inflation and deflation phase during the LVOM, and (c) time to stabilization of lung volume after a pressure change. We also quantified thoraco-abdominal synchrony in lung aeration during the LVOM. Changes in lung aeration were quantified using respiratory inductance plethysmography (RIP), as we have previously demonstrated that this may better reflect changes in lung aeration than markers for oxygenation [9, 10].

## Methods

The present study is a secondary analysis of prospectively collected data from a previous study in subjects < 18 years of age who were managed with HFOV for acute respiratory failure [10]. The indication for HFOV was set by the attending physician based on institutional clinical guidelines. Subjects were enrolled after written informed consent from either parents or legal caretakers. The Institutional Review Board of the hospital approved the study.

### Study population

Included were subjects with acute onset of lung disease, < 18 years of age who were managed with HFOV for acute respiratory failure [10]. Subjects with a gestational age < 40 weeks, with congenital or acquired paralysis of the diaphragm, uncorrected congenital heart disorder, severe pulmonary hypertension or open thorax or abdomen after surgery and those with status asthmaticus were excluded.

### HFOV lung volume optimization strategy

**Fig. 1:**
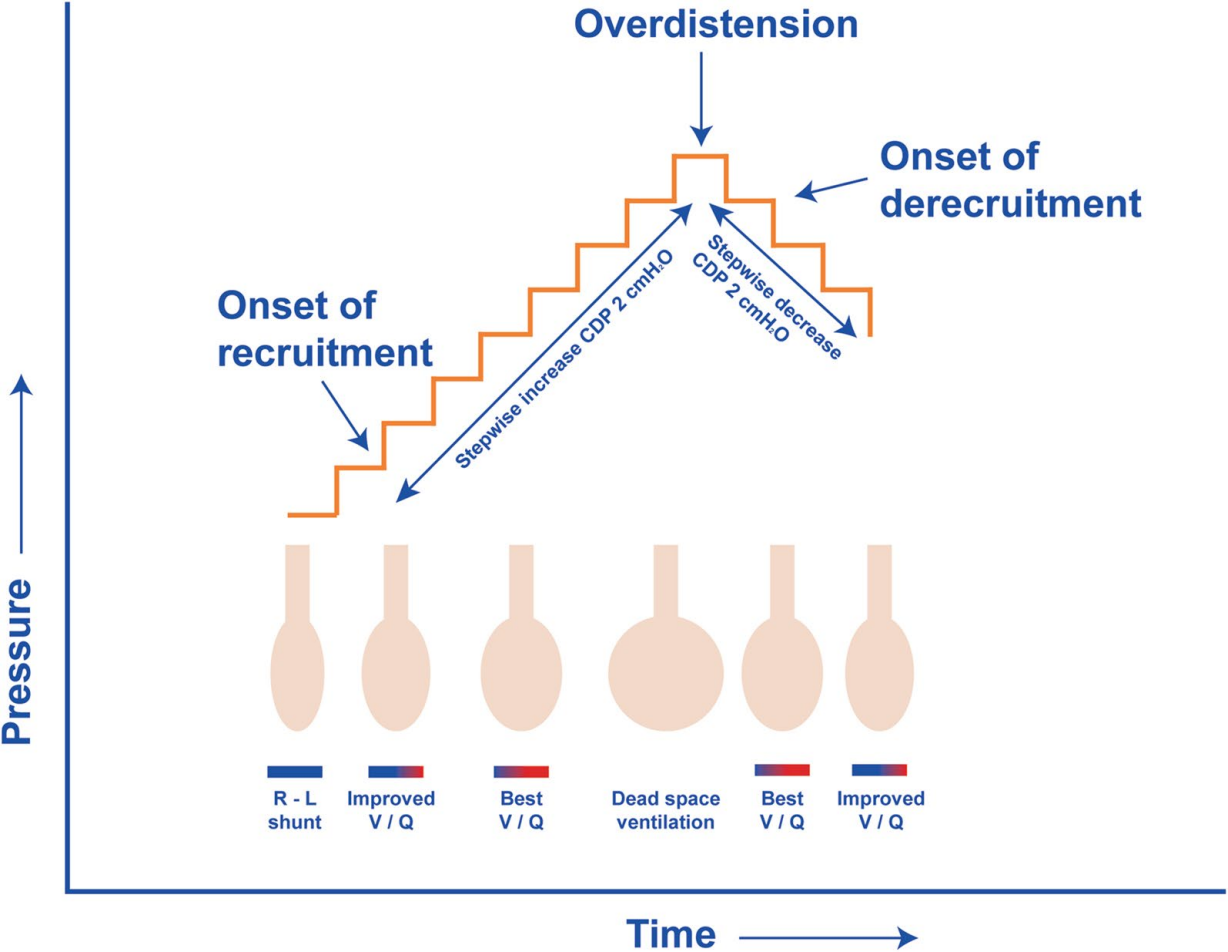
Graphic representation of the inflation phase of the lung volume optimization maneuvre. During the incremental pressure titration lung recruitment is monitored using the transcutaneous oxygen saturation (SpO_2_). First, pressure is increased stepwise by 2 cmH_2_O until overdistension occurs. Then, pressure is reduced stepwise by 2 cmH_2_O. *CDP* continuous distending pressure, *R–L* shunt right-to-left shunting

Patients were oscillated per an institutional-based protocol which defines HFOV criteria, the LVOM and titration of HFOV settings according to the evolving physiological needs of the subject as has been published previously [8]. In brief, the LVOM describes an individualized staircase incremental–decremental CDP titration, performed immediately after transitioning to HFOV using the SensorMedics 3100A/B oscillator (SensorMedics, Yorba Linda, CA, USA). Initial oscillator settings irrespective of age or bodyweight included frequency (F) 10–12 Hz, CDP 3–5 cmH_2_O above mean airway pressure (mPaw) on conventional mechanical ventilation (CMV), power setting targeted at a proximal pressure amplitude (∆*P*_proximal_ 70–90 cmH_2_O), inspiratory time 33% and bias flow 20–40 L/min. The CDP was increased by 2 cmH_2_O every 3–5 min until the onset of lung recruitment was identified by increases in SpO_2_ (CDP_recruitment_). The stepwise increase of the CDP was continued until no further improvement in oxygenation and/or sudden decrease in mean arterial blood pressure (mABP) occurred during two consecutive increments identifying the onset of overdistention of the lung (CDP_hyperinflation_). Subsequently, mPaw was stepwise decreased every 3–5 min by 2 cmH_2_O until the SpO_2_ decreased again during two consecutive increments indicating lung derecruitment (CDP_derecruitment_) (Fig. 1). The LVOM was repeated to CDP_hyperinflation_ with setting the “optimal” CDP 2 cmH_2_O above the CDP_derecruitment_. During the LVOM, power remained constant, and F was only decreased in patients with severe increasing hypercapnia resulting in acidosis (pH < 7.25) so that Δ*P*_proximal_ could reflect changes in compliance.

### Data acquisition

CDP and ∆P_proximal_ were measured at the airway opening using the Bicore II™ respiratory monitor (Vyaire, Yorba Linda, CA, USA). RIP signals were sampled at 200 Hz using two elastic bands (Nox Medical, Reykjavik, Iceland) connected to the Bicore II™ (Vyaire, Yorba Linda, CA, USA) and stored for offline analysis. Data acquisition was done using a custom-built Polybench software module (Applied Biosignals, Weener, Germany).

### Data analysis

Time, CDP, and RIP signals were sampled and stored at 200 Hz. Offline, analysis was performed in Matlab (the MathWorks Inc. (2022), Natick, MA, USA). An additional signal was created using an exponential moving average filter on the troughs of the pressure signal. Time was calculated based on the sampling frequency and sample index. The pressure and Respiratory Inductance (RIP) signals were filtered using a low-pass fourth-order Butterworth filter with a cutoff frequency of 0.05 (used for removal of high-frequency noise). In addition, RIP signals were high-pass filtered, with a cutoff frequency of 4 Hz to remove movement artifacts and cardiac signals. Data preceding recruitment (defined as pressure signal without HFOV pressure variation) were omitted from analysis. Data were sliced into epochs for each change in CDP. CDP pressure changes were identified using flank detection on the low-pass filtered pressure signal, and steps with duration less than 60 s or a difference from the preceding step smaller than 0.75 cmH_2_O were discarded. For each epoch, mean airway pressure was calculated and rounded to the nearest integer value. High- and low-pass filtered RIP signals were recombined.

For each subject, we normalized the CDP pressure changes by setting the pressure at which the maneuver started (initial and lowest pressure at 0% (CDP_start_) and the last (highest) pressure (CDP_max_) during the incremental phase of the maneuver at 100%. Subsequently, all steps of both the inflation part and deflation part of the recruitment were normalized. We only used the first incremental–decremental phase of the LVOM.

The amplitude (difference in RIP signal) at each pressure change (∆RIP) was determined. To determine the response in lung volume changes immediately after initiation of HFOV, we determined at each normalized CDP pressure change if ∆RIP showed a positive (i.e., increase in RIP signal) or a negative (i.e., decrease in RIP signal) direction during the inflation and deflation phase of the LVOM. The percentage of positive and negative ∆RIP was calculated and plotted against the normalized CDP. To determine the balance between recruitment and derecruitment within each pressure change, we arbitrarily divided each pressure change into periods of 10 s during the inflation and deflation phase of the LVOM. For each 10-s period, we determined if the RIP showed a positive (i.e., increase in RIP signal) or a negative (i.e., decrease in RIP signal) direction, calculated the overall ratio of positive and negative periods and these plotted against the normalized CDP to determine if there were differences in this ratio depending on the position of the pressure–volume relationship.

To determine the time to stable lung volume after a pressure change, we calculated the time constant (tau) using a previously described model consuming that the change in pressure behaves like first-order system. The time constant per epoch was calculated on the summed filtered RIP signals following determination of slope (upward or downward) using methods described earlier by Niemann et al. [12]. We assume that lung volume changes resulting from changes in mean airway pressure can be modeled as the response for the first-order system with a unit step input, given by

$${\text{RIP}}\left(t\right)=g\left(1-{e}^{-t \left/ \tau \right.}\right),$$

with g the gain per step and t the time since pressure change [12]. Tau represents the time 63.2% of the maximal gain of lung volume is achieved. Tau values higher than 300 s were excluded, because CDP was stepwise increased/decreased every 3–5 min. Pressure changes that did not follow this first-order system were classified by visual inspection in three groups (decrease, increase or no increase or decrease of the RIP signal). Both tau and visual classified steps were plotted against the normalized CDP.

To determine thoraco-abdominal synchrony in lung aeration during the LVOM, we calculated the phase angle between the thoracic and abdominal RIP signal and plotted them against the normalized CDP for both the inflation and deflation phase of the LVOM. The phase difference between the recombined chest and abdominal recombined signals was computed using fast Fourier transform (FFT) following a Hanning window of each signal, yielding a phase for both chest and abdominal signal. The difference between both phases yields the phase difference in radians, which then was recomputed to degrees.

### Statistical analysis

Categorical data are presented as percentage (%) of total and continuous data as median [25–75] interquartile range (IQR) when assumptions of normality were not met. The Kruskal–Wallis test was used to analyze continuous data and linear regression was used to analyze changes in RIP signal against normalized CDP using GraphPad version 10 (GraphPad Software, San Diego, CA, USA). *p* values less than 0.05 were accepted as statistically significant.

## Results

RIP data of 51 subjects (median age 3.5 [1.7–13.3] months) were available for analysis, yielding an aggregate of 408 steps in the incremental and 243 steps in the decremental phase of the LVOM. Study population characteristics, last CMV settings before HFOV and HFOV settings are summarized in Table 1. In the cohort, 24.4% suffered from severe and 61.0% from moderate PARDS. Almost all subjects (86.3%) had direct lung injury related to infections.

**Table 1:** Characteristics of the study population

Number of patients	51
Patient’s characteristics	
Male/female (%)	53/47
Age (months)	3.5 (1.7–13.3)
< 12 months (%)	74.5
12–24 months (%)	3.9
> 48 months (%)	21.6
Weight (kg)	6.0 (4.5–9.3)
PRISM II (24h) score	
Pulmonary admission diagnosis (%)	86.3
PARDS (%)	80.4
Mild (%)	14.6
Moderate (%)	61.0
Severe (%)	24.4
Duration of MV (days)	6.6 (4.9–10.6)
Mortality (%)	2.0
Ventilation characteristics on CMV	
PIP (cmH_2_O)	31 (30–34)
PEEP (cmH_2_O)	8 (6–9)
FiO_2_	0.5 (0.4–0.8)
SpO_2_/FiO_2_ ratio on CMV	190 (121–243)
OI on CMV	10.3 (8.3–16.1)
pH on CMV	7.30 (7.23–7.35)
pCO_2_ on CMV (mmHg)	59 (53–64)
Ventilation characteristics on HFOV	
CDP start	20 (18–22)
CDP max	34 (32–37)
CDP end	26 (24–28)

*PRISM* pediatric risk of mortality, *PARDS* pediatric acute respiratory distress syndrome, *MV* mechanical ventilation, *CMV* conventional mechanical ventilation, *PIP* peak inspiratory pressure, *PEEP* positive end-expiratory pressure, *FiO*_*2*_ fraction of inspired oxygen, *SpO*_*2*_ transcutaneous oxygen saturation, *OI* oxygenation index, *HFOV* high-frequency oscillatory ventilation, *CDP* continuous distending pressure

### Overall recruitment versus derecruitment during the LVOM

**Fig. 2:**
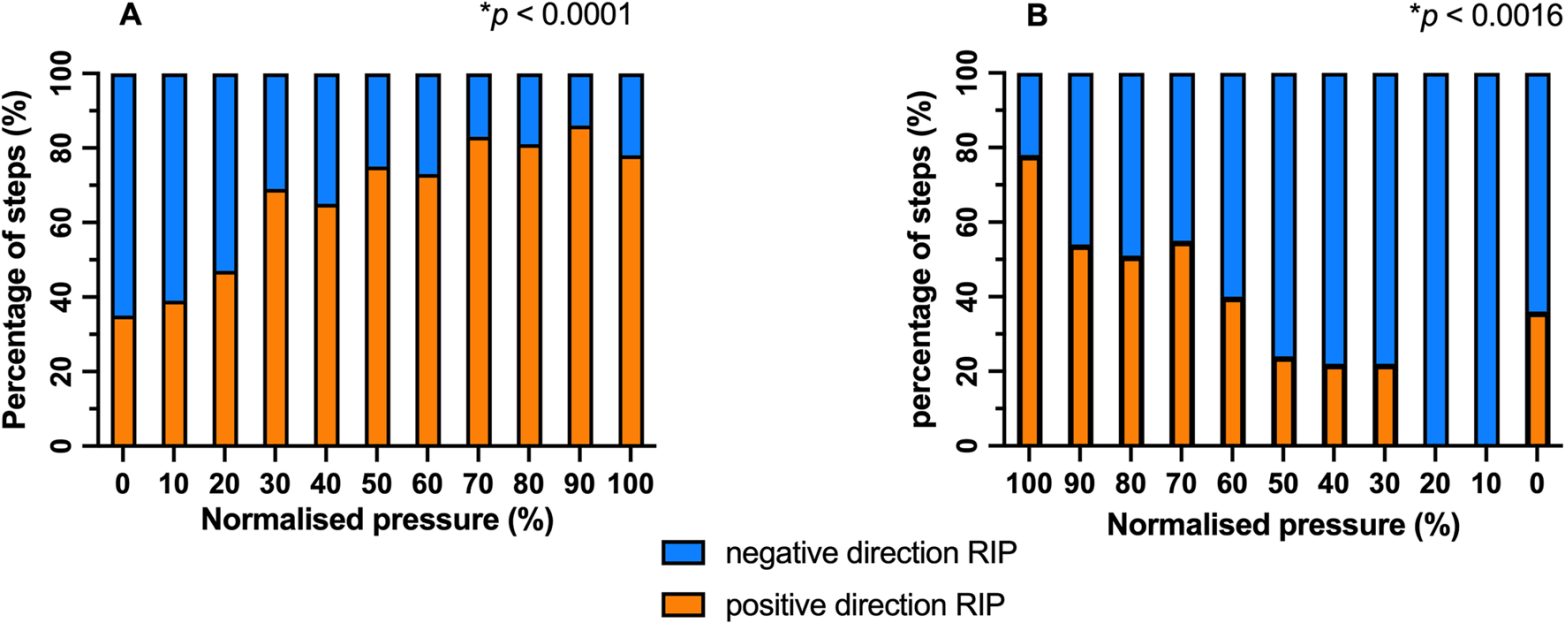
Distribution of the percentage of pressure changes with an increase in RIP signal suggestive for lung recruitment and a decrease in RIP signal suggestive for lung derecruitment plotted against the normalized continuous distending pressure (CDP) during the inflation (**A**) and deflation (**B**) phase of the lung volume optimization maneuver (LVOM). RIP respiratory inductance plethysmography; LVOM lung volume optimization maneuver. *p < 0.05

First, we explored the overall lung behavior during the incremental and decremental phase of the LVOM. The mean initial CDP at start of the LVOM was 20 (18–22) cmH_2_O. In 65% a negative direction of the total RIP signal was observed, indicative for alveolar collapse (Fig. 2A). The percentage of CDP changes with positive direction of the RIP signal increased statistically (*p* < 0.0001) with increasing pressure. Changes in total RIP signal showed that CDP changes with positive and negative direction occurred during the inflation phase of the LVOM, but there were CDP steps with positive direction at normalized CDP ≥ 30%. During the deflation phase, there was a significant increase in the percentage of steps with a negative direction of the RIP signal (*p* < 0.0016) (Fig. 2B). We observed that already at 90% of CDP_max_ almost half of all steps the RIP signal had a negative direction, suggestive of lung derecruitment. Similar results were observed when the rib cage and abdominal RIP signals were analyzed separately (data not shown).

### Recruitment versus derecruitment within a step

**Fig. 3:**
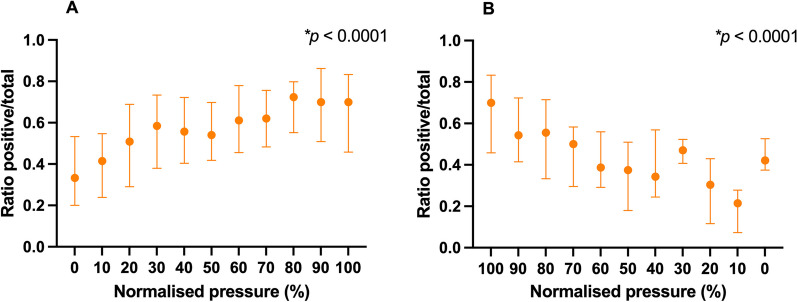
Distribution within a pressure change of the ratio (displayed as median [IQR] of the number of 10-s periods with an increase in RIP signal suggestive for lung recruitment and a decrease in RIP signal suggestive for lung derecruitment plotted against the normalized continuous distending pressure (CDP) over the total number of 10-s periods during the inflation (**A**) and deflation (**B**) phase of the lung volume optimization maneuver (LVOM). *RIP* respiratory inductance plethysmography, *CDP* continuous distending pressure, *LVOM* lung volume optimization maneuver. *p < 0.05

Next, we studied within each pressure change lung behavior during the LVOM. During the inflation phase, we observed a significant increase in the fraction of periods with a positive direction of the RIP signal (Fig. 3A). At HFOV start, the ratio was 0.33 (0.20, 053), meaning that 33% of the 10-s periods had a positive RIP direction. The fraction stabilized from 80% of CDP_max_ and was 0.70 (0.46, 0.83) at the end of the recruitment (CDP_max_). During the deflation phase, the fraction decreased below 0.40 from 30% of CDP_max_ (Fig. 3B).

### Time constant calculation

**Fig. 4:**
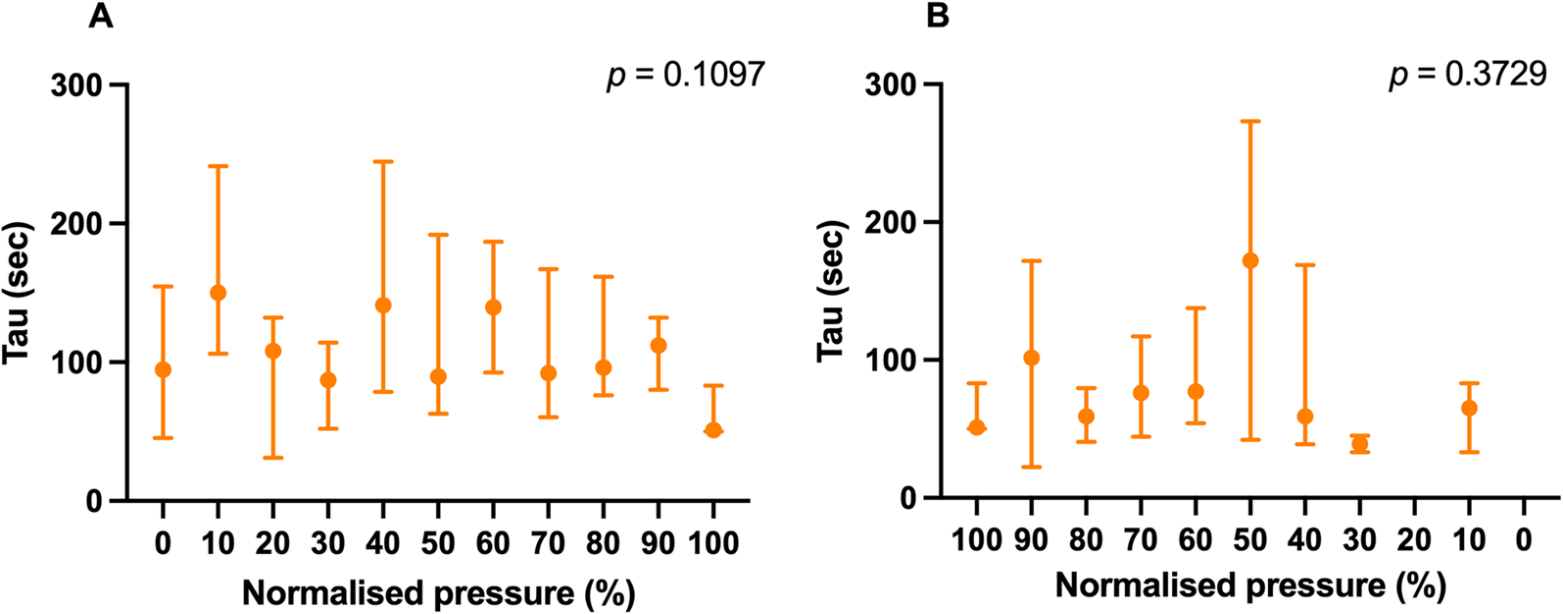
Distribution of the time constant (tau) plotted against the normalized CDP during the inflation (**A**) and deflation (**B**) phase of the LVOM. Data are displayed as median [IQR]. *RIP* respiratory inductance plethysmography, *CDP* continuous distending pressure, *LVOM* lung volume optimization maneuver. *p < 0.05

The time constant could only be calculated in 26.2% of all pressure changes during the inflation and in 21.4% during the deflation phase of the LVOM. Additional file 1: Fig. S1 summarizes the distribution of the percentage of steps where it was possible to calculate the time constant plotted against normalized CDP. The possibility of calculating the time constant was independent from CDP. Inability to calculate the time constant was due to lack of stabilization of the RIP signal (i.e., it remained increasing or decreasing at the end of a pressure change), or it did not change in any direction (Additional file 1: Fig. S2), which was dependent on the normalized pressure. We observed a heterogeneity in the time constant plotted against normalized CDP, albeit it was not significantly dependent on the CDP during the inflation or deflation phase of the LVOM (Fig. 4A, B).

### Thoraco-abdominal synchrony of the RIP signal

**Fig. 5:**
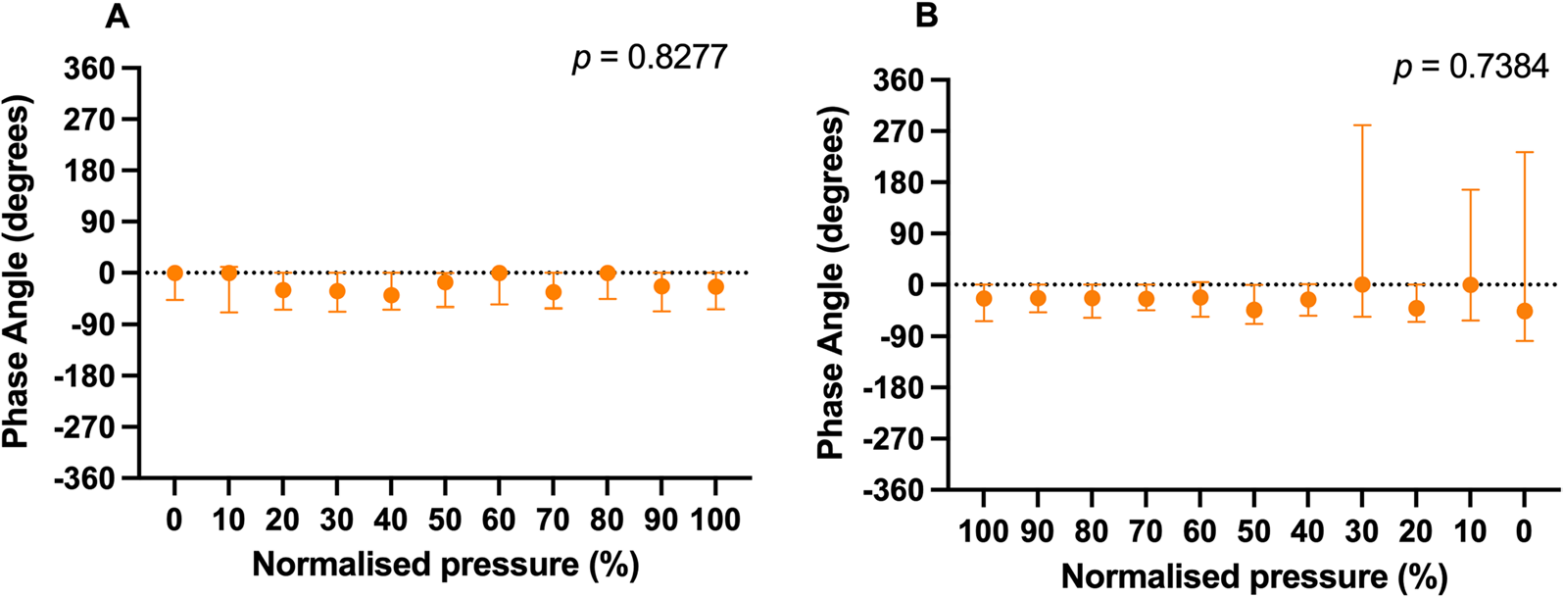
Distribution of the phase angle plotted against the normalized CDP during the inflation (**A**) and deflation (**B**) phase of the LVOM. Data are displayed as median [IQR]. *RIP* respiratory inductance plethysmography, *CDP* continuous distending pressure, *LVOM* lung volume optimization maneuver. *p < 0.05

The phase angle plotted against the normalized CDP remained constant throughout the inflation and deflation phase of the LOVM (Fig. 5A, B).

Subanalyses stratified by age and pARDS severity are summarized in Additional file 1: Figs. E3–E8.

## Discussion

To our best of knowledge, this is the first pediatric study describing lung behavior during a staircase incremental–decremental lung volume optimization maneuver in subjects with moderate-to-severe acute respiratory failure including PARDS on HFOV. The main findings of our study can be summarized as follows: we found that in most subjects the initial CDP resulted in lung derecruitment and that several incremental pressure changes were required before significant lung recruitment occurred. Also, at low normalized CDP there was within a pressure change more derecruitment than recruitment. On the inflation limb of the pressure–volume loop there was both lung recruitment and derecruitment, and on the deflation limb derecruitment occurred very rapidly. The time constant could only be calculated in a small number of pressure changes, suggesting that lung recruitment is heterogeneous and time-consuming during the LVOM.

At present, it is unclear what the best LVOM in pediatric HFOV constitutes. In the absence of supportive data for any LVOM or the other, international pediatric guidelines recommend using a staircase incremental–decremental pressure titration as LVOM [2, 3]. The LVOM used in our unit and analyzed in the present study included pressure changes by 2 cmH_2_O every 3–5 min from an initial CDP 3–5 cmH_2_O above mPaw on CMV until the onset of lung recruitment was identified by increases in SpO_2_ during the inflation phase, and a 2 cmH_2_O decrease every 3–5 min until the SpO_2_ decreased, indicative of lung derecruitment. The results from our study indicate that further refinements to this LVOM approach are necessary. First, the initial CDP chosen may have been too low as there was a negative direction of the RIP signal suggestive of lung derecruitment in more than half of the pressure changes during the first 20% of the LVOM. Significant onset of lung recruitment occurred only afterward. This signifies the need for a higher initial CDP (i.e., > 5 cmH_2_O) when transitioning to HFOV. Second, the time between each pressure step in our clinical algorithm is arbitrarily defined at 3–5 min, but our study shows that this may have been too short. In the great majority of pressure changes, we could not calculate the time constant. This is because we found much heterogeneity in changes in RIP direction which were also dependent on the position of the pressure volume loop. At low inflation pressures during the inflation phase, most changes in RIP signal were negative and did not stabilize, whereas in another group of pressure changes, there was either a continuous positive or negative RIP direction change without stabilization. From the pressure changes that allowed calculation of the time constant, we found that the time between two pressure changes needed to be at least 5 min during the inflation deflation phase of the LVOM. While there are no pediatric data to compare our findings with, a study in pre-term neonates with infant respiratory distress syndrome reported stabilization of lung volume after a pressure change within 5 min whereas in 13 predominantly term or ex-preterm infants on rescue HFOV it took more than 10 min [13, 14]. The latter findings support our observation, although it should be mentioned that our population has much more heterogeneous lung disease. The time constant on the deflation limb was shorter than on the inflation limb, which was also observed in neonates [13, 14].

A sustained inflation (SI) was used as LVOM in adults randomized to HFOV in the Oscillation for Acute Respiratory Distress Syndrome (ARDS) Treated Early (OSCILLATE) trial [15]. During this maneuver, a pressure of 40 cmH_2_O was delivered for 40 s. Our study was not designed to compare different LVOMs, but based on our present findings it may be postulated that an SI would unlikely result in an appropriate lung aeration, although this has to be explored in further studies using imaging techniques.

One of the at least theoretical benefits of a staircase incremental–decremental pressure change LVOM is that it allows for oscillation on the deflation limb of the pressure volume loop, making use of hysteresis of the lungs. In support of this are the observations of both lung recruitment and derecruitment on the inflation limb of the pressure–volume loop, but at the same time, on the deflation limb we also found significant derecruitment occurring very rapidly, indicating that at least in some subjects there was only a small hysteresis.

There are some limitations to our study that need to be addressed. First, RIP measures cross-sectional changes of the thorax and does not quantify lung volume unless calibrated, but it has been found to correlate with changes in lung volume in experimental [16, 17] and neonatal clinical studies [6, 18]. Second, it may be argued that we did not fully recruit the lung which may have affected some of our results. Indeed, we found that the ratio of RIP signals with a positive direction during each pressure change increased during the inflation phase of the LVOM to a maximum of 0.7, surmising that there was still a degree of derecruitment. Theoretically, if the lung was fully recruited than the ratio would be 1. Also, we did not observe changes in the phase angle. It may be hypothesized that if a lung is fully recruited, the phase angle would change because the change in RIP signal would mainly come from the abdomen because of depression of the diaphragm. On the other hand, we also found that early on when mapping the deflation limb of the pressure–volume loop there was in approximately half of the pressure changes an increase in RIP signal, suggesting that the compression of alveoli due to overdistension of other alveoli at high inflation pressures was reduced when inflation pressures were reduced. This question can only be addressed in future studies making use of imaging techniques such as electrical impedance tomography (EIT).

## Conclusions

We have found significant heterogeneity in lung behavior during a staircase incremental–decremental LVOM in pediatric patients on HFOV, suggesting the need for higher initial inflation pressures when transitioning from CMV and a longer time between pressure changes to allow for equilibration. Further work is necessary to expand on these findings, using imaging techniques to optimize and individualize HFOV titration.

### Supplementary Information

**Additional file 1: Figure S1.** Distribution of the percentage of pressure changes in which the time constant could be calculated plotted against the normalized CDP during the inflation (A) and deflation (B) phase of the LVOM. RIP respiratory inductance plethysmography; CDP continuous distending pressure; LVOM lung volume optimalization maneuver. * denotes *p* < 0.05. **Figure S2.** Distribution of pressure steps in which no time constant could be calculated because of continuous increase, continuous decrease or no change in any direction of the RIP plotted against the normalized CDP during the inflation (A) and deflation (B) phase of the LVOM. RIP respiratory inductance plethysmography; CDP continuous distending pressure; LVOM lung volume optimalization maneuver. * denotes *p* < 0.05. **Figure S3.** Stratified by age. Distribution of the percentage of pressure changes with an increase in RIP signal suggestive for lung recruitment and a decrease in RIP signal suggestive for lung derecruitment plotted against the normalized continuous distending pressure (CDP) during the inflation (left side) and deflation (right side) phase of the lung volume optimization maneuver (LVOM). Only percentage-groups including more than 3 measurements are included. Upper panel: subjects < 6 months (A inflation phase, B deflation phase), middle panel subjects 6–24 months (C inflation phase, D deflation phase) and lower panel: subjects > 24 months inflation phase (E inflation phase, F deflation phase). RIP respiratory inductance plethysmography; LVOM lung volume optimalization maneuver. * denotes *p* < 0.05. **Figure S4.** Stratified by age. Distribution within a pressure change of the ratio (displayed as median [IQR] of the number of 10-second periods with an increase in RIP signal suggestive for lung recruitment and a decrease in RIP signal suggestive for lung derecruitment plotted against the normalized continuous distending pressure (CDP) over the total number of 10-second periods during the inflation (left side) and deflation (right side) phase of the lung volume optimization maneuver (LVOM). Only percentage-groups including more than 3 measurements are included. Upper panel: < 6 months (A inflation phase, B deflation phase), middle panel 6–24 months (C inflation phase, D deflation phase) and lower panel: > 24 months inflation phase (E inflation phase, F deflation phase). RIP respiratory inductance plethysmography; CDP continuous distending pressure; LVOM lung volume optimalization maneuver. * denotes *p* < 0.05. **Figure S5.** Stratified by age. Distribution of the phase angle plotted against the normalized CDP during the inflation (left side) and deflation (right side) phase of the LVOM. Data are displayed as median [IQR]. Upper panel: < 6 months (A inflation phase, B deflation phase), middle panel 6 – 24 months (C inflation phase, D deflation phase) and lower panel: > 24 months inflation phase (E inflation phase, F deflation phase). RIP respiratory inductance plethysmography; CDP continuous distending pressure; LVOM lung volume optimalization maneuver. * denotes *p* < 0.05. **Figure S6.** Stratified by pARDS severity. Distribution of the percentage of pressure changes with an increase in RIP signal suggestive for lung recruitment and a decrease in RIP signal suggestive for lung derecruitment plotted against the normalized continuous distending pressure (CDP) during the inflation (left side) and deflation (right side) phase of the lung volume optimization maneuver (LVOM). Only percentage-groups including more than 3 measurements are included. Upper panel: mild pARDS (A inflation phase, B deflation phase), middle panel moderate pARDS (C inflation phase, D deflation phase) and lower panel: severe pARDS (E inflation phase, F deflation phase). RIP respiratory inductance plethysmography; LVOM lung volume optimalization maneuver. * denotes *p* < 0.05. **Figure S7.** Stratified by pARDS severity. Distribution within a pressure change of the ratio (displayed as median [IQR] of the number of 10-second periods with an increase in RIP signal suggestive for lung recruitment and a decrease in RIP signal suggestive for lung derecruitment plotted against the normalized continuous distending pressure (CDP) over the total number of 10-second periods during the inflation (left) and deflation (right) phase of the lung volume optimization maneuver (LVOM). Upper panel: mild pARDS (A inflation phase, B deflation phase), middle panel moderate pARDS (C inflation phase, D deflation phase) and lower panel: severe pARDS (E inflation phase, F deflation phase). RIP respiratory inductance plethysmography; CDP continuous distending pressure; LVOM lung volume optimalization maneuver. * denotes *p* < 0.05. **Figure S8.** Distribution of the phase angle plotted against the normalized CDP during the inflation (left side) and deflation (right side) phase of the LVOM. Data are displayed as median [IQR]. Upper panel: mild pARDS (A inflation phase, B deflation phase), middle panel moderate pARDS (C inflation phase, D deflation phase) and lower panel: severe pARDS (E inflation phase, F deflation phase). RIP respiratory inductance plethysmography; CDP continuous distending pressure; LVOM lung volume optimalization maneuver. * denotes *p* < 0.05.

## Data Availability

The datasets used and analyzed during the current study are available from the corresponding author on reasonable request.

## Abbreviations

CMV: Conventional mechanical ventilation
CDP: Continuous distending pressure
CDP_derecruitment_: CDP point representing onset of derecruitment
CDP_hyperinflation_: CDP point representing onset of hyperinflation
CDP_max_: Highest CDP point during the LVOM
CDP_recruitment_: CDP point representing onset of lung recruitment
CDP_start_: CDP point representing the start of the LVOM
CMV: Conventional mechanical ventilation
HFOV: High-frequency oscillatory ventilation
F: Frequency
FiO_2_: Fraction of inspired oxygen
HFOV: High-frequency oscillatory ventilation
IQR: Interquartile range
LVOM: Lung volume optimization maneuvers
mABP: Mean arterial blood pressure
mPaw: Mean airway pressure
MV: Mechanical ventilation
OI: Oxygenation index
PEEP: Positive end-expiratory pressure
PARDS: Pediatric acute respiratory distress syndrome
PIP: Peak inspiratory pressure
∆*P*_proximal_: Proximal pressure amplitude
PRISM: Pediatric risk of mortality
∆RIP: The amplitude (difference in RIP signal) at each pressure change
RIP: Respiratory inductance plethysmography
SpO_2_: Transcutaneous oxygen saturation
