# Supplementary material for: Lung behavior during a staircase high-frequency oscillatory ventilation recruitment maneuver
Source: Intensive Care Med Exp. 2024 Apr 25;12:42. doi: 10.1186/s40635-024-00623-w (PMC11045697; doi:10.1186/s40635-024-00623-w)

# **Lung behavior during a staircase high-frequency oscillatory ventilation recruitment maneuver**

**Online data supplement**

Pauline de Jager, MD (1)

Alette A. Koopman, MSc (1)

Dick G. Markhorst, MD PhD (2)

Martin C.J. Kneyber, MD PhD FCCM (1,3)

Figure E1

Distribution of the percentage of pressure changes in which the time constant could be calculated plotted against the normalized CDP during the inflation (A) and deflation (B) phase of the LVOM.

RIP respiratory inductance plethysmography; CDP continuous distending pressure; LVOM lung volume optimalization maneuver.

* denotes *p* < 0.05.

Figure E2

Distribution of pressure steps in which no time constant could be calculated because of continuous increase, continuous decrease or no change in any direction of the RIP plotted against the normalized CDP during the inflation (A) and deflation (B) phase of the LVOM.

RIP respiratory inductance plethysmography; CDP continuous distending pressure; LVOM lung volume optimalization maneuver.

* denotes *p* < 0.05.

Figure E3

Stratified by age. Distribution of the percentage of pressure changes with an increase in RIP signal suggestive for lung recruitment and a decrease in RIP signal suggestive for lung derecruitment plotted against the normalized continuous distending pressure (CDP) during the inflation (left side) and deflation (right side) phase of the lung volume optimization maneuver (LVOM). Only percentage-groups including more than 3 measurements are included.

Upper panel: subjects < 6 months (A inflation phase, B deflation phase), middle panel subjects 6 – 24 months (C inflation phase, D deflation phase) and lower panel: subjects > 24 months inflation phase (E inflation phase, F deflation phase).

RIP respiratory inductance plethysmography; LVOM lung volume optimalization maneuver.

* denotes *p* < 0.05.


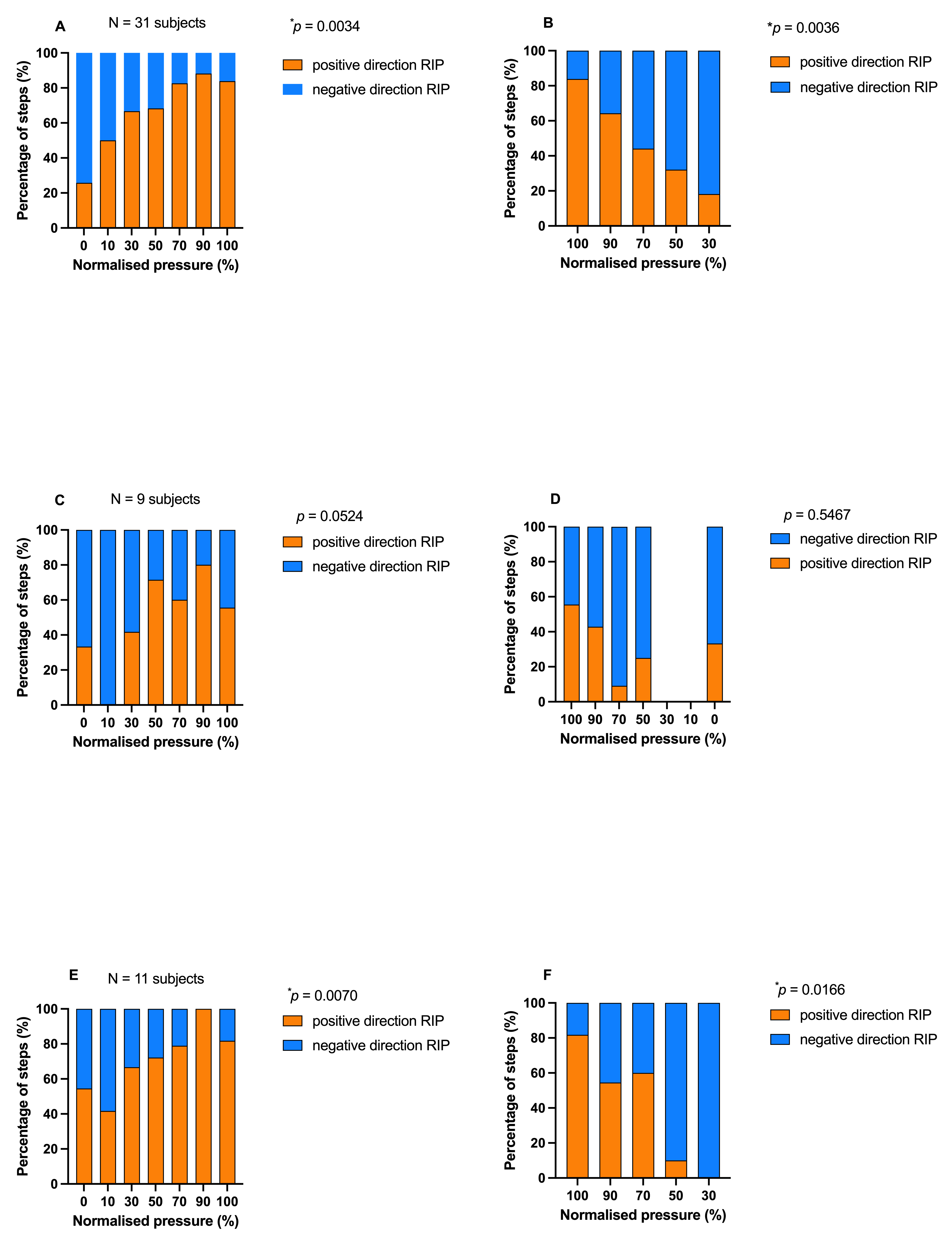


Figure E4

Stratified by age. Distribution within a pressure change of the ratio (displayed as median [IQR] of the number of 10-second periods with an increase in RIP signal suggestive for lung recruitment and a decrease in RIP signal suggestive for lung derecruitment plotted against the normalized continuous distending pressure (CDP) over the total number of 10-second periods during the inflation (left side) and deflation (right side) phase of the lung volume optimization maneuver (LVOM). Only percentage-groups including more than 3 measurements are included.

Upper panel: < 6 months (A inflation phase, B deflation phase), middle panel 6 – 24 months (C inflation phase, D deflation phase) and lower panel: > 24 months inflation phase (E inflation phase, F deflation phase).

RIP respiratory inductance plethysmography; CDP continuous distending pressure; LVOM lung volume optimalization maneuver.

* denotes *p* < 0.05.


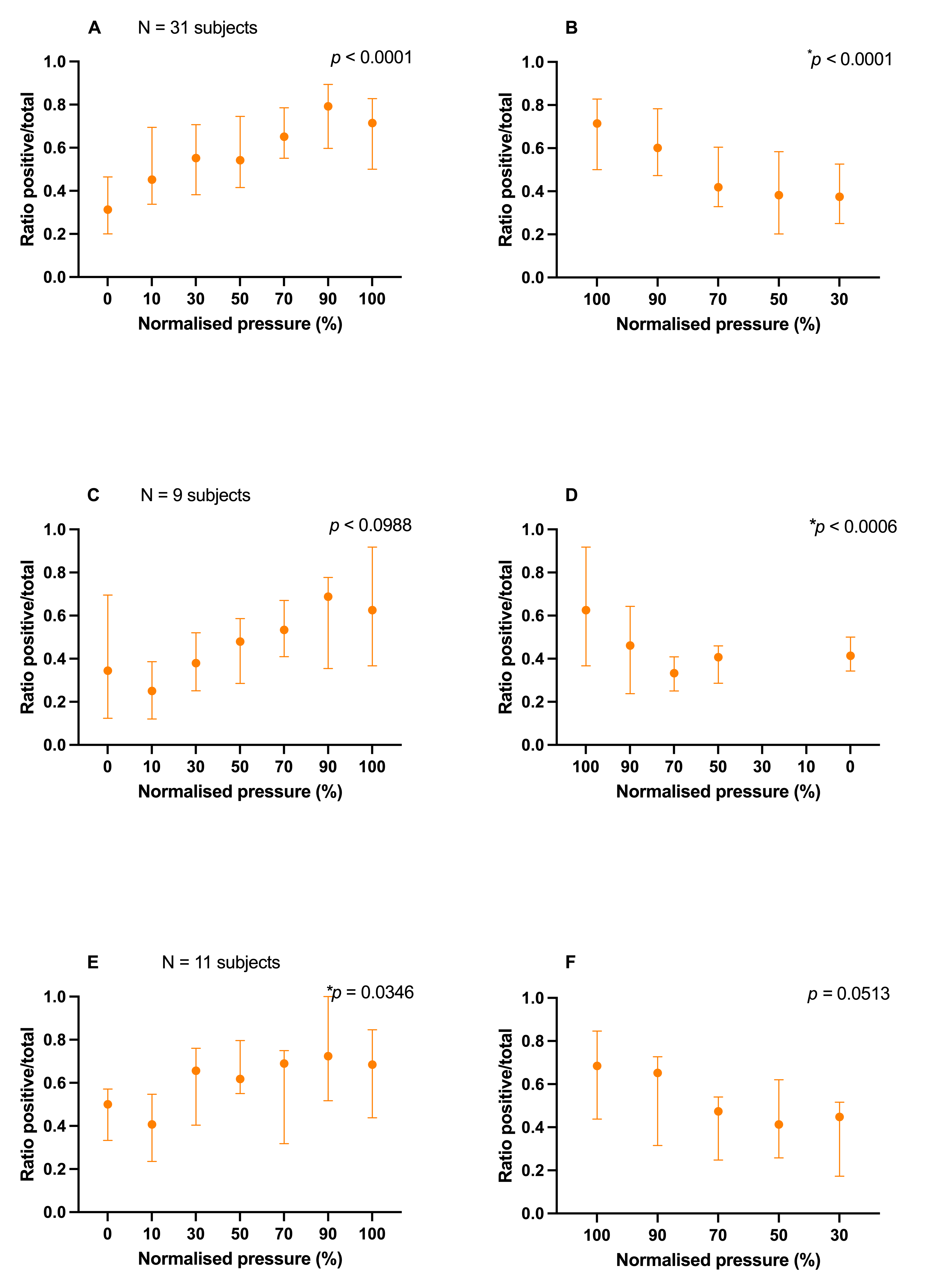


Figure E5

Stratified by age.

Distribution of the phase angle plotted against the normalized CDP during the inflation (left side) and deflation (right side) phase of the LVOM. Data are displayed as median [IQR].

Upper panel: < 6 months (A inflation phase, B deflation phase), middle panel 6 – 24 months (C inflation phase, D deflation phase) and lower panel: > 24 months inflation phase (E inflation phase, F deflation phase).

RIP respiratory inductance plethysmography; CDP continuous distending pressure; LVOM lung volume optimalization maneuver.

* denotes *p* < 0.05.


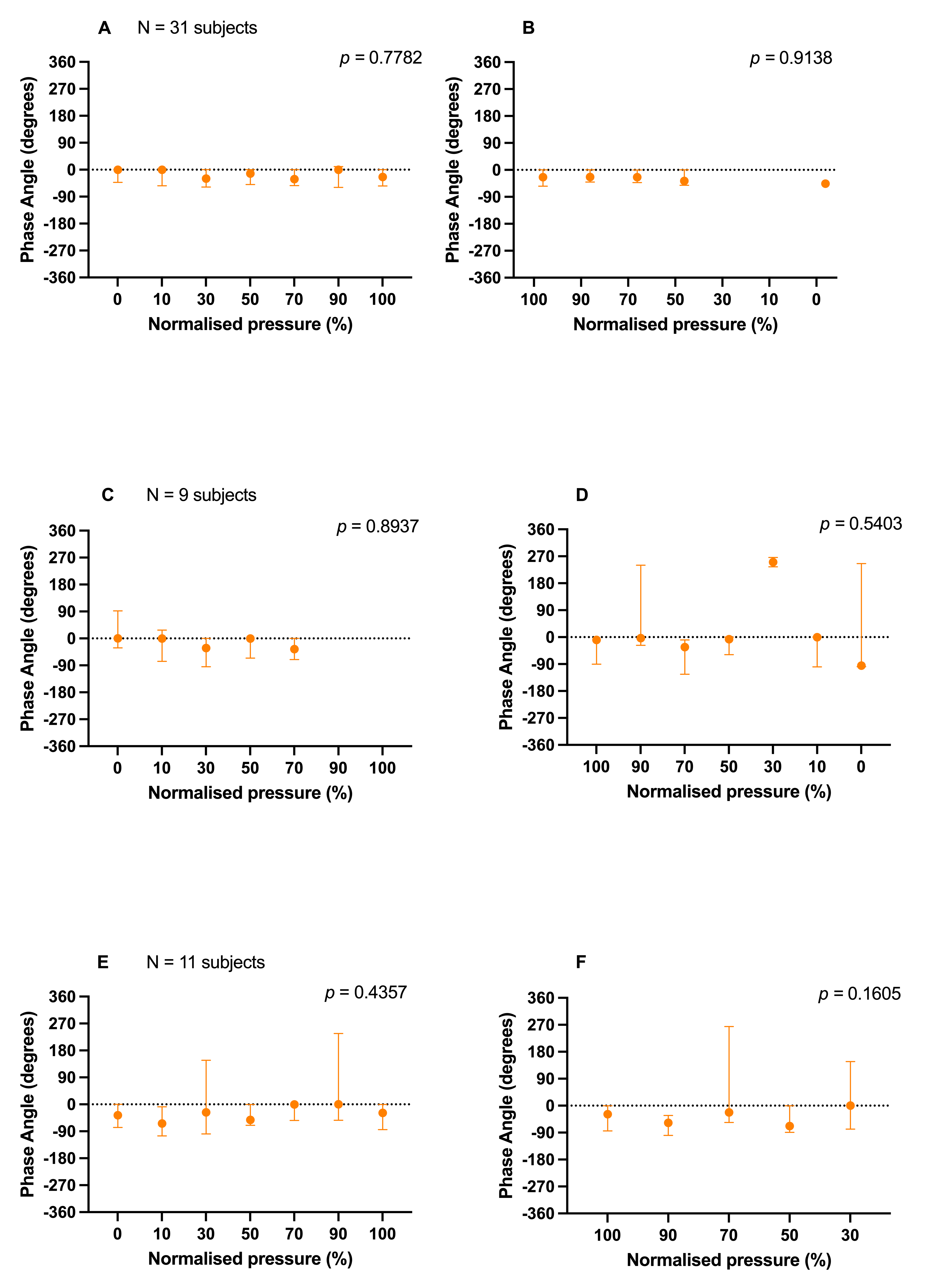


Figure E6

Stratified by pARDS severity.

Distribution of the percentage of pressure changes with an increase in RIP signal suggestive for lung recruitment and a decrease in RIP signal suggestive for lung derecruitment plotted against the normalized continuous distending pressure (CDP) during the inflation (left side) and deflation (right side) phase of the lung volume optimization maneuver (LVOM). Only percentage-groups including more than 3 measurements are included.

Upper panel: mild pARDS (A inflation phase, B deflation phase), middle panel moderate pARDS (C inflation phase, D deflation phase) and lower panel: severe pARDS (E inflation phase, F deflation phase).

RIP respiratory inductance plethysmography; LVOM lung volume optimalization maneuver.

* denotes *p* < 0.05.


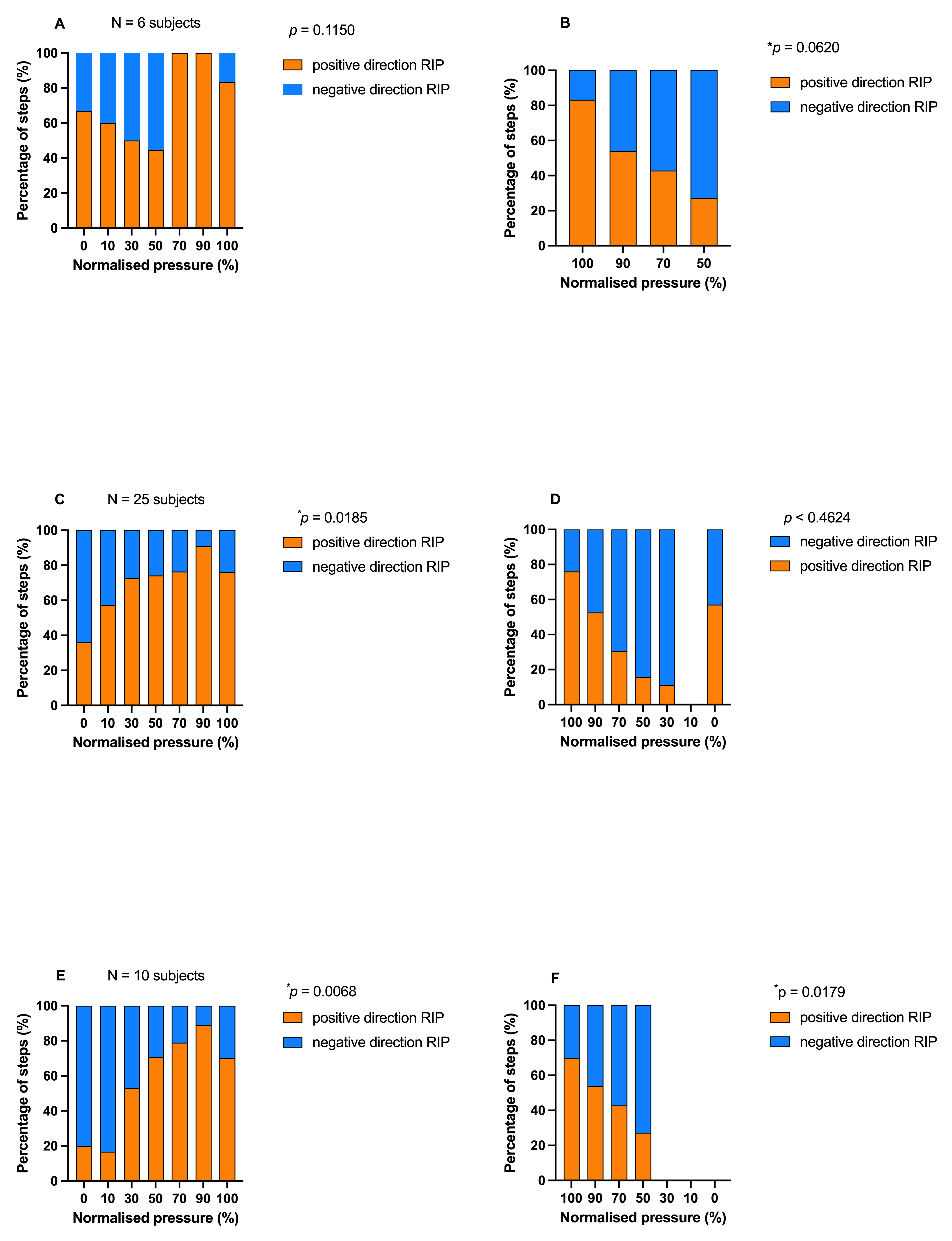


Figure E7

Stratified by pARDS severity.

Distribution within a pressure change of the ratio (displayed as median [IQR] of the number of 10-second periods with an increase in RIP signal suggestive for lung recruitment and a decrease in RIP signal suggestive for lung derecruitment plotted against the normalized continuous distending pressure (CDP) over the total number of 10-second periods during the inflation (left) and deflation (right) phase of the lung volume optimization maneuver (LVOM).

Upper panel: mild pARDS (A inflation phase, B deflation phase), middle panel moderate pARDS (C inflation phase, D deflation phase) and lower panel: severe pARDS (E inflation phase, F deflation phase).

RIP respiratory inductance plethysmography; CDP continuous distending pressure; LVOM lung volume optimalization maneuver.

* denotes *p* < 0.05.


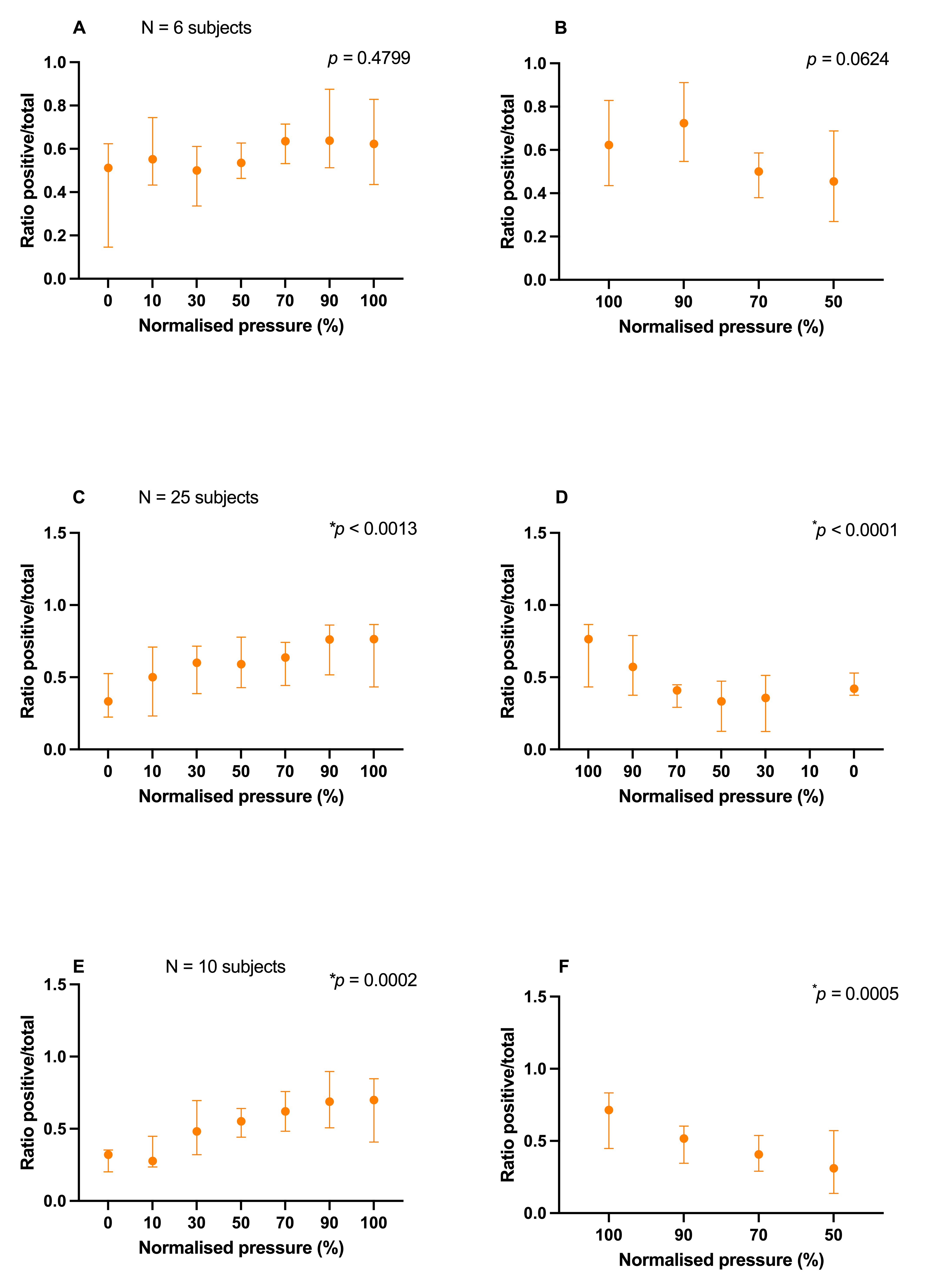


Figure E8

Distribution of the phase angle plotted against the normalized CDP during the inflation (left side) and deflation (right side) phase of the LVOM. Data are displayed as median [IQR].

Upper panel: mild pARDS (A inflation phase, B deflation phase), middle panel moderate pARDS (C inflation phase, D deflation phase) and lower panel: severe pARDS (E inflation phase, F deflation phase).

RIP respiratory inductance plethysmography; CDP continuous distending pressure; LVOM lung volume optimalization maneuver.

* denotes *p* < 0.05.


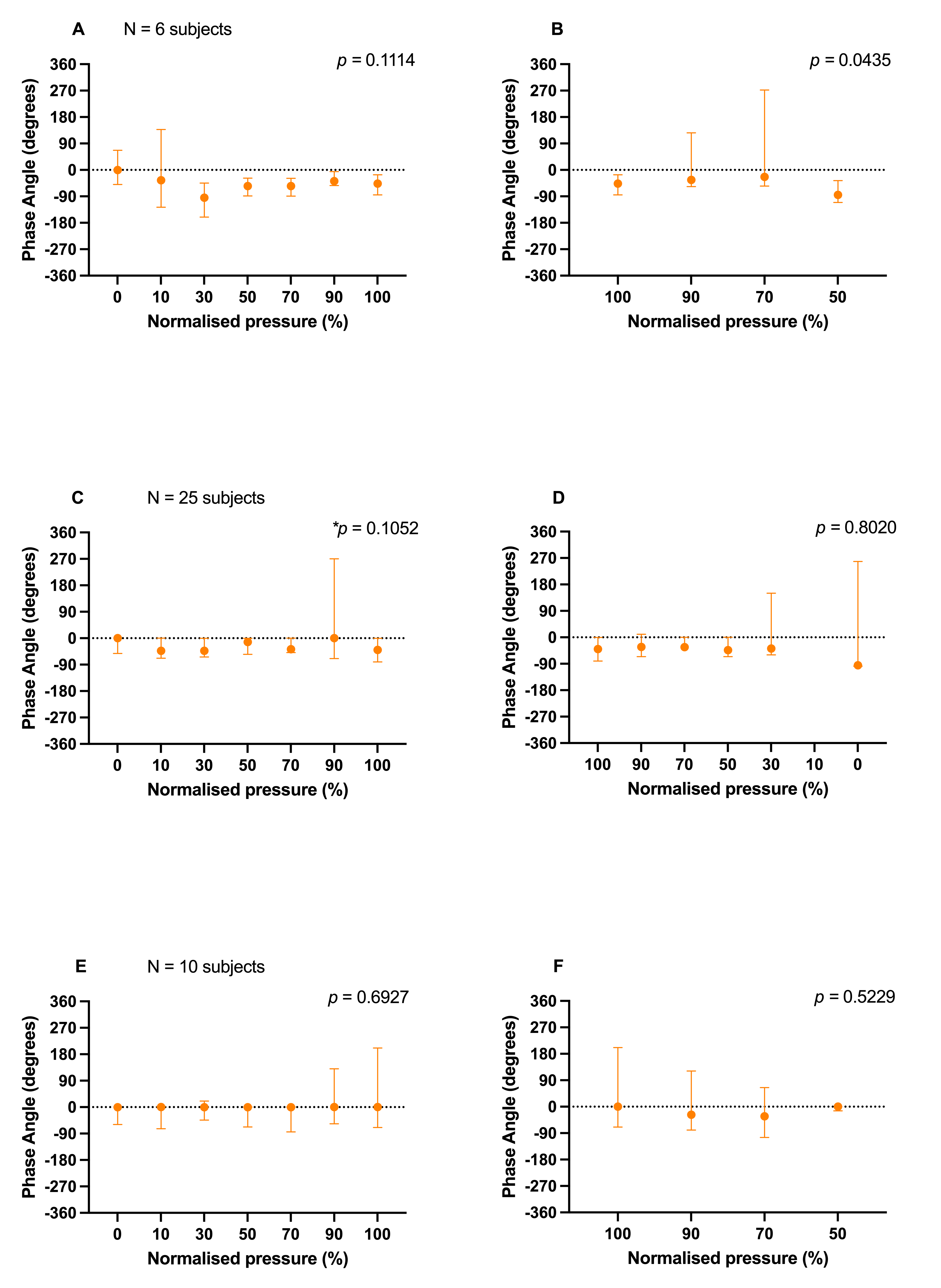

Supplement: Supplementary file 1 — Additional file 1: Figure S1. Distribution of the percentage of pressure changes in which the time constant could be calculated plotted against the normalized CDP during the inflation (A) and deflation (B) phase of the LVOM. RIP respiratory inductance plethysmography; CDP continuous distending pressure; LVOM lung volume optimalization maneuver. * denotes p < 0.05. Figure S2. Distribution of pressure steps in which no time constant could be calculated because of continuous increase, continuous decrease or no change in any direction of the RIP plotted against the normalized CDP during the inflation (A) and deflation (B) phase of the LVOM. RIP respiratory inductance plethysmography; CDP continuous distending pressure; LVOM lung volume optimalization maneuver. * denotes p < 0.05. Figure S3. Stratified by age. Distribution of the percentage of pressure changes with an increase in RIP signal suggestive for lung recruitment and a decrease in RIP signal suggestive for lung derecruitment plotted against the normalized continuous distending pressure (CDP) during the inflation (left side) and deflation (right side) phase of the lung volume optimization maneuver (LVOM). Only percentage-groups including more than 3 measurements are included. Upper panel: subjects < 6 months (A inflation phase, B deflation phase), middle panel subjects 6–24 months (C inflation phase, D deflation phase) and lower panel: subjects > 24 months inflation phase (E inflation phase, F deflation phase). RIP respiratory inductance plethysmography; LVOM lung volume optimalization maneuver. * denotes p < 0.05. Figure S4. Stratified by age. Distribution within a pressure change of the ratio (displayed as median [IQR] of the number of 10-second periods with an increase in RIP signal suggestive for lung recruitment and a decrease in RIP signal suggestive for lung derecruitment plotted against the normalized continuous distending pressure (CDP) over the total number of 10-second periods during the inflation (lef [file 40635_2024_623_MOESM1_ESM.docx]
